# Prevalence of idiopathic normal pressure hydrocephalus - a pilot study in Jämtland, Sweden

**DOI:** 10.1186/2045-8118-12-S1-O55

**Published:** 2015-09-18

**Authors:** Catherine Michelle Rosell, Johanna Andersson, Karin Kockum, Otto Lilja-Lund, Lars Söderström, Katarina Laurell

**Affiliations:** 1 Department of Pharmacology and Clinical Neuroscience, Unit of Research, Education and Development, Östersund, Umeå University, Sweden; 2 Unit of Research, Education and Development, Östersund Hospital, Region Jämtland Härjedalen, Sweden

## Introduction

This pilot study was conducted to describe the prevalence, age and gender distribution of idiopathic normal pressure hydrocephalus (iNPH) in Jämtland, a Swedish county.

## Methods

A total of 200 people over the age of 65 were randomly selected from the Swedish population registry and sent a simple questionnaire with questions regarding gait and balance disturbance, continence and/or cognitive impairment, i.e. the cardinal symptoms of iNPH. A total of 67 of the responding participants, with and without symptoms of iNPH were then selected to undergo a CT scan of the brain and clinical examination. INPH was classified as “probable”, “possible” or “unlikely” according to modified European guidelines. Hellströms iNPH scale (1) was used to measure the severity of symptoms in the domains of gait, neuropsychology, balance and continence.

## Results

Out of 66 people, 8 (12 %) received the diagnosis “probable”, 8 “possible” (12%) and 50 “unlikely” (66 %) iNPH, which indicates a prevalence of 4% for the diagnosis “probable” iNPH in the total population of 200. There was a statistical difference in gender distribution weighted towards men but not between the age groups 80+ vs <80, a result most likely due to the lower number of participants over 80 years of age. Those diagnosed as unlikely iNPH, had a significantly higher mean iNPH score (87), corresponding to less symptoms, than those with probable iNPH (62) (p= 0.01).

## Conclusion

The prevalence of probable iNPH in the population of 65 years and older was found to be 4 % with a higher proportion of men than women being diagnosed. Although the severity of symptoms was related to the iNPH diagnosis, classification according to the European guidelines revealed some need for revision. The results of this pilot study will be confirmed in a larger sample of 1000 persons.
